# *Salvia miltiorrhiza* (SM) Injection Ameliorates Iron Overload-Associated Cardiac Dysfunction by Regulating the Expression of DMT1, TfR1, and FP1 in Rats

**DOI:** 10.1155/2021/6864723

**Published:** 2021-05-26

**Authors:** Yuanyuan Zhang, Yucong Xue, Bin Zheng, Xue Han, Donglai Ma, Zhihong Ma, Shengjiang Guan, Yonggang Gao, Ziliang Li, Li Chu

**Affiliations:** ^1^School of Pharmacy, Hebei University of Chinese Medicine, Shijiazhuang 050200, Hebei, China; ^2^Hebei Key Laboratory of Integrative Medicine on Liver-Kidney Patterns, Shijiazhuang 050200, Hebei, China; ^3^School of Basic Medicine, Hebei University of Chinese Medicine, Shijiazhuang 050200, Hebei, China

## Abstract

Previous studies have found that *Salvia miltiorrhiza* (SM) injection have a protective effect on the iron overloaded (IO) heart. However, the mechanisms are not completely known. In the present study, we investigated the underlying mechanisms based on the iron transport-related proteins. The rats were randomly divided into five groups: control, IO group, low-dose SM group, high-dose SM group, and deferoxamine control group. Iron dextran was injected to establish the IO model. After 14 days of treatment, cardiac histological changes were observed by hematoxylin and eosin (H&E) staining. Iron uptake-related proteins divalent metal transporter-1 (DMT-1), transferrin receptor-1 (TfR-1), and iron export-related proteins ferroportin1 (FP1) in the heart were detected by Western blotting. The results showed that SM injection decreased cardiac iron deposition, ameliorated cardiac function, and inhibited cardiac oxidation. Most important of all, SM injection downregulated the expression of DMT-1 and TfR-1 and upregulated FP1 protein levels compared with the IO group. Our results indicated that reducing cardiac iron uptake and increasing iron excretion may be one of the important mechanisms of SM injection reducing cardiac iron deposition and improving cardiac function under the conditions of IO.

## 1. Introduction

Iron is vital to the maintenance of normal cellular structure, function, and metabolic activity. Iron overload (IO) is a clinical problem usually secondary to diseases such as hereditary hemochromatosis [1] and myelodysplastic syndromes [2] or diseases that require long-term repeated blood transfusions, such as thalassemia syndrome [3]. The heart is one of the main target organs for iron deposition under conditions of IO. When levels of labile iron exceed the capacity of cardiomyocytes, these labile cellular irons can activate a large-scale oxidative stress response that leads to oxidative damage [4]. IO cardiomyopathy, caused by cardiac iron deposition, can progress to dilated cardiomyopathy and eventually lead to heart failure [5].

Under physiological conditions, cardiomyocytes maintain cardiac iron homeostasis by regulating iron uptake/storage in order to maintain normal cardiac function. A growing number of studies indicate that transferrin receptors (TfRs) and divalent metal transporter (DMT) may be two major routes for iron entry into cardiomyocytes during IO conditions [6]. In addition, ferroportin 1 (FP1), which is expressed in the heart, liver, and other tissues in humans and mice, has been regarded as exporter of cellular iron [7]. Based on these previous findings, this study investigated the regulative effects of *Salvia miltiorrhiza* (SM) on the three iron transporter proteins under IO conditions.

SM, also named as Danshen, is a traditional Chinese herbal medicine belonging to the *Labiatae* family that possesses obvious cardiovascular regulation effects. SM injection, for which the injection substance consists of a water extraction from SM roots and rhizomes, has been used to treat chest tightness from coronary heart disease and angina pectoris in clinical settings. According to previous studies, SM injection can ameliorate heart [8, 9], liver [10, 11], and kidney [12] injuries caused by IO through inhibiting iron accumulation, antioxidation, antiapoptosis, and antifibrosis. However, the mechanisms by which SM injection reduces iron accumulation are not well known. In this study, we investigated the regulative effects of SM injection on iron transport-related proteins to elucidate the underlying mechanisms with SM reducing cardiac iron.

## 2. Materials and Methods

### 2.1. Drugs and Reagents

The *Salvia miltiorrhiza* (SM) injection (1.5 g SM crude drug per milliliter) was purchased from Shenlong Pharmaceutical Co., Ltd. (Jiangsu, China, approval number: Z32020161, lot number: 16102114). Iron dextran and desferrioxamine (DFO) were purchased from Pharmacosmos A/S and Novartis Pharma, respectively. Unless otherwise stated, other chemicals were purchased from Sigma Chemical (Saint Louis, MO, USA).

### 2.2. Animals and Experimental Design

Sixty adult male Sprague-Dawley (SD) rats (220–260 g) were purchased from Hebei Medical University, and the rats were housed in an automatic temperature (∼22°C) and humidity (∼50%) room with a 12 h light/dark cycle and given food and water *ad libitum*. All animal handling procedures were in accordance with the Guidelines of Animal Experiments from the Committee of Medical Ethics, National Health Department of China. This study was approved by the Ethics Committee for Animal Experiments of the Hebei University of Chinese Medicine (approval number: 1805024; approval date: May 7, 2018).

After one week of acclimation, the rats were randomly divided into five groups (*n* = 12 per group): control, iron overload (IO), low-dose-SM (L-SM), high-dose-SM (H-SM), and desferrioxamine (DFO, which is a chelating agent used to remove excess iron) group. Rats in the control group were treated with 0.1 mL/10 g body weight of saline daily by intraperitoneal injection (IP). Rats in the IO group were injected with 50 mg/kg of iron dextran daily. Rats in the H-SM, L-SM, and DFO groups were injected at 8 : 00 a.m. with the same dose of iron dextran and injected at 6 : 00 p.m. with 6 g/kg/day SM, 3 g/kg/day SM, and 100 mg/kg/day DFO, respectively. Food intake and activities of all rats were observed daily and the entire experimental period lasted 14 days.

### 2.3. Main Constituent Analysis of SM Injection

**Figure 1:**
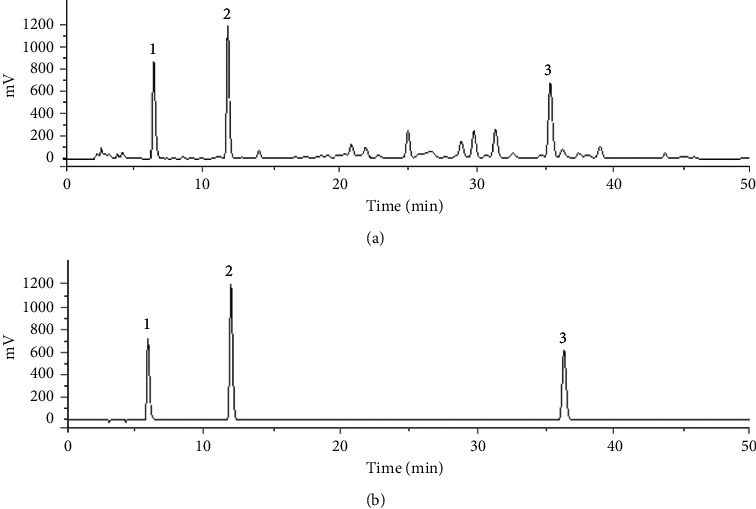
HPLC–UV profiles of Danshen injection (a). The mixed standard solution of Danshen (b). Peaks represent: 1, salvianolic acid A; 2, protocatechuic aldehyde; 3, salvianolic acid B.

Three main constituents (salvianolic acid A, protocatechuic aldehyde, and salvianolic acid B) of the SM injection were detected by high performance liquid chromatography with ultraviolet (HPLC-UV) and the chromatograms were presented in Figure 1. The conditions and procedures have been described previously [8, 13].

### 2.4. Acquisition of Hemodynamic Parameters

After 14 days of administration, the rats were anesthetized by intraperitoneal injection of sodium pentobarbital (50 mg/kg), and a micropressure sensor catheter (Chengdu Instrument Co., Ltd., Chengdu, China) was implanted into their left ventricles through their right carotid arteries. Heart rate (HR), left ventricular systolic pressure (LVSP), left ventricular end-diastolic pressure (LVEDP), and maximum rising and falling rates of left intraventricular pressure (±dp/dt_max_) were monitored and then recorded and analyzed after 10 min of stabilization using an MS4000U-1C analysis system for quantitative recording of biological signals (Guangzhou Science and Technology Co., Ltd., Guangzhou, China). At the end of the experiment, blood was taken from the femoral arteries, and the serum was centrifuged for further analysis. Heart samples were quickly removed and fixed in 4% paraformaldehyde solution or frozen in liquid nitrogen for later analysis.

### 2.5. Hematoxylin and Eosin (H&E) and Prussian Blue Staining

The fixed cardiac tissues were embedded in paraffin and cut into 4 *μ*m sections. The sections were then deparaffined, dehydrated using graded ethanol, and stained according to the manufacturers' protocols with H&E for histopathological analysis or with Prussian blue for cardiac iron accumulation analysis. Image-Pro Plus software was used to analyze Prussian blue-stained samples, and values were expressed as a percentage of area that was positive (% positive area = positive area/sum area ∗ 100%).

### 2.6. Detection of Cardiac Iron by Flame Atomic Absorption Spectroscopy (FAAS)

The frozen cardiac tissue samples were dried at 65°C for 24 h, and then the dried samples were weighed and ashed at 500°C for 12–16 h. The ashed samples were digested by hydrochloric acid (10 M, 1 mL) and nitric acid (6 M, 3 mL) heating to a slow boil, then hydrogen peroxide was added (4% w/v, 2 drops) boiling over 4∼6 h until only 0.5 mL of liquid remained. Deionized water was added to the concentrated samples (totally 20 mL) and absorbance readings were performed at 248.3 nm using Spectra AA-10 varian spectrophotometer (Agilent Technologies, USA). Standard curves for iron were prepared according to the standards.

### 2.7. Assessment of Creatine Kinase (CK) and Lactate Dehydrogenase (LDH) Activity in Serum

Total serum CK and LDH activities were determined at 37°C using commercially available kits (BoiSino Bio-Technology and Science, Inc., Beijing, China). All procedures were performed according to the manufacturer's protocol.

### 2.8. Detection of Superoxide Dismutase (SOD) Activity and Malondialdehyde (MDA) Content in Cardiac Tissue

Cardiac tissue homogenate (100 mg tissue per mL of 50 mM phosphate buffer) was prepared using a tissue homogenizer, after which the homogenates were centrifuged and the supernatants were used for analyses. SOD activity and MDA content in heart tissues were estimated using commercially available kits (Jian Cheng Biological Engineering Institute, Nanjing, China).

### 2.9. Measurement of Iron Transport-Related Proteins in Cardiac Function

Three iron transport-related proteins (DMT-1, TfR-1, and FP1) were detected by Western blotting. Frozen heart tissue samples were homogenized with 400 *μ*L lysis buffer/20 mg tissue and then centrifuged at 12,000 r/min for 10 min, after which the supernatant was collected. Total proteins were loaded and separated on a 10% SDS-PAGE (sodium dodecyl sulfate polyacrylamide gel electrophoresis) gel and then transferred to a nitrocellulose membrane. Blocking was performed with 5% skimmed milk powder in phosphate buffer saline (PBS), and then the membranes were incubated with primary antibodies overnight at 4°C. The membrane was washed three times (10 min each) to remove uncombined primary antibodies, and then it was incubated with secondary antibodies at room temperature for 90 min. After the membrane was washed three times to remove unbound secondary antibodies, the membrane was exposed and scanned by the Gel imaging analysis system (UVP, USA), and the gray value was automatically measured by the system. The primary antibodies included anti-DMT1 antibody (ab55735, Abcam), anti-transferrin receptor antibody [MEM-189] (ab1086, Abcam), and anti-ferroportin/SLC40A1 antibody (ab58695, Abcam).

### 2.10. Data Analysis

Data are expressed as the mean ± standard error of the mean (SEM). Statistically significant differences were identified using a one-way analysis of variance (ANOVA) followed by Tukeyʼs post hoc multiple comparison test. Differences were considered statistically significant at *P* < 0.05. The statistical analysis software, Origin 7.5, was used for all analyses.

## 3. Results

### 3.1. Contents Analysis of SM Injection

The three main constituent of SM injection was detected by HPLC-UV shown in Figure 1. The actual concentrations of the salvianolic acid A is 2.15 mg/mL, protocatechuic aldehyde is 0.44 mg/mL, and salvianolic acid B is 1.01 mg/mL.

### 3.2. Histomorphology Changes

**Figure 2:**
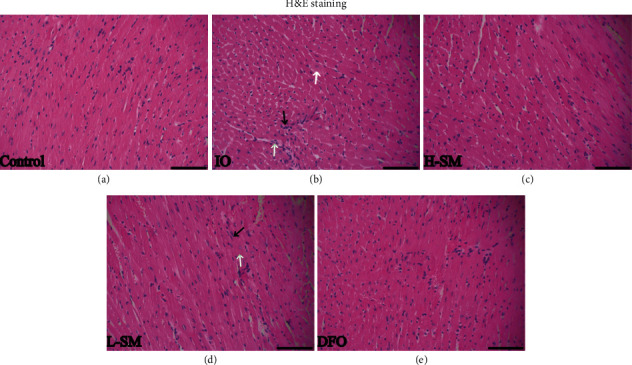
Effects of SM injection on morphological changes in heart tissue. Samples were obtained from the control group (saline), iron overload (IO) group (iron dextran 50 mg/kg), high-dose SM group (H-SM, iron dextran 50 mg/kg + SM 6 g/kg), low-dose SM group (L-SM, iron dextran 50 mg/kg + SM 3 g/kg), and DFO group (iron dextran 50 mg/kg + DFO 100 mg/kg). The cell nucleus swelled (black arrow) together with myocardial fibers, which resulted in atrophy and rupture (white arrows) as shown by H&E staining (magnification 200x, scale bar = 100 *μ*m).

H&E staining was used to observe cardiac histomorphology changes in each group. The IO group exhibited obvious swelling of nuclei and atrophy and rupture of myocardial fibers. In mice that received SM injection and DFO treatment, the nuclear swelling was reduced and the myocardial fibers were arranged regularly compared with the IO group (Figure 2).

### 3.3. Reducing the Cardiac Iron Deposition

**Figure 3:**
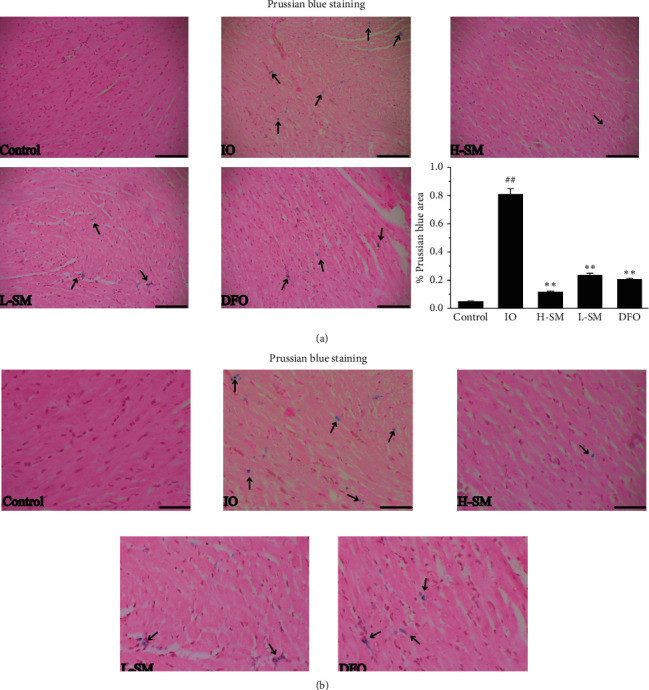
Effects of SM injection on iron deposition changes in heart tissue. (a) The blue reactions (black arrow) on the sections represent deposited iron (magnification 200x, scale bar = 100 *μ*m). The percentage of Prussian blue-positive area (Prussian blue area (%) = (positive area/sum area) ∗ 100%) in each group was quantified. Values are mean ± S.E.M. ^##^*P* < 0.01 vs. control group; ^*∗∗*^*P* < 0.01 vs. IO group. (b) The same sections with figure A from each group were taken as a magnification of 400x, scale bar = 50 *μ*m.

Prussian blue staining was used to observe cardiac iron deposition. The results showed that large Prussian blue-positive areas were observed in the IO cardiac tissues, which indicated obvious iron deposition. In the SM and DFO treatment rats, Prussian blue-positive areas reduced significantly compared with the IO group (Figure 3).

**Figure 4:**
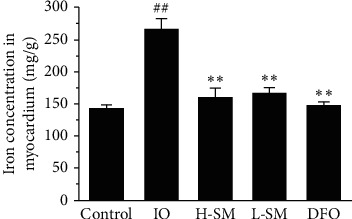
Effects of SM injection on total cardiac iron concentration. Values are mean ± S.E.M. ^##^*P* < 0.01 vs. control group; ^*∗∗*^*P* < 0.01 vs. IO group.

The FAAS results indicated the changes of total iron in cardiac tissues. As shown in Figure 4, cardiac iron concentration of the IO model rats was significantly increased (*P* < 0.01) compared with that in the control rats. However, compared with the IO group, cardiac iron concentration was decreased (*P* < 0.01) in the SM injection and DFO treatment groups.

### 3.4. Improving the Cardiac Function

Some hemodynamic parameters were recorded to assess left ventricular systolic and diastolic functions. As shown in Table 1, maximum rising and falling rates of left intraventricular pressure (±dp/dt_max_) decreased and diastolic pressure increased in the IO group compared to the control group (*P* < 0.05). This indicates that IO can induce left ventricular systolic and diastolic dysfunction. In the SM and DFO groups, systolic pressure and ±dp/dt_max_ increased and diastolic pressure decreased compared to the IO group (*P* < 0.05), which suggests that SM can significantly improve the functions of contraction and diastole of left ventricle.

**Figure 5:**
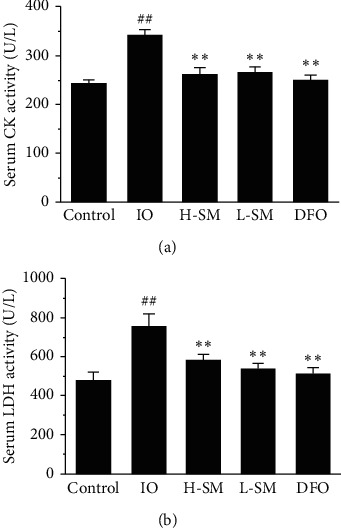
Effects of SM injection on serum CK and LDH activity. Values are mean ± SEM. ^##^*P* < .01 vs. control group; ^*∗∗*^*P* < .01 vs. IO group.

In addition, the serum activities of the myocardial enzymes CK and LDH were assessed to evaluate cardiac function. In the IO group, CK and LDH activity increased ∼141.67% and ∼152.64%, respectively, compared to the control group (*P* < .01). CK and LDH activity were reduced by ∼27.1% and ∼32.4%, respectively, in the H-SM group compared to the IO group (*P* < .01) (Figure 5).

### 3.5. Decreasing the Cardiac Oxidative Stress Levels

**Figure 6:**
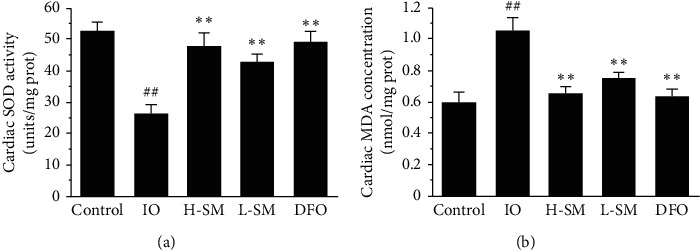
Effects of SM injection on SOD activity and MDA concentration in cardiac homogenate. Values are mean ± SEM. ^##^*P* < .01 vs. control group; ^*∗∗*^*P* < .01 vs. IO group.

The antioxidant enzyme SOD and the oxidation product MDA were assayed to observe changes in oxidative stress among the iron overloaded and SM- and DFO-treated groups. As shown in Figure 6, SOD activity was inhibited and MDA concentration increased in the IO group compared to the control group. In the SM and DFO groups, SOD activity increased and MDA concentration declined simultaneously. These results indicate that reducing cardiac oxidative stress levels may be a mechanism by which SM ameliorates cardiac function.

### 3.6. Regulating the Expression of Iron Transport-Related Proteins

**Figure 7:**
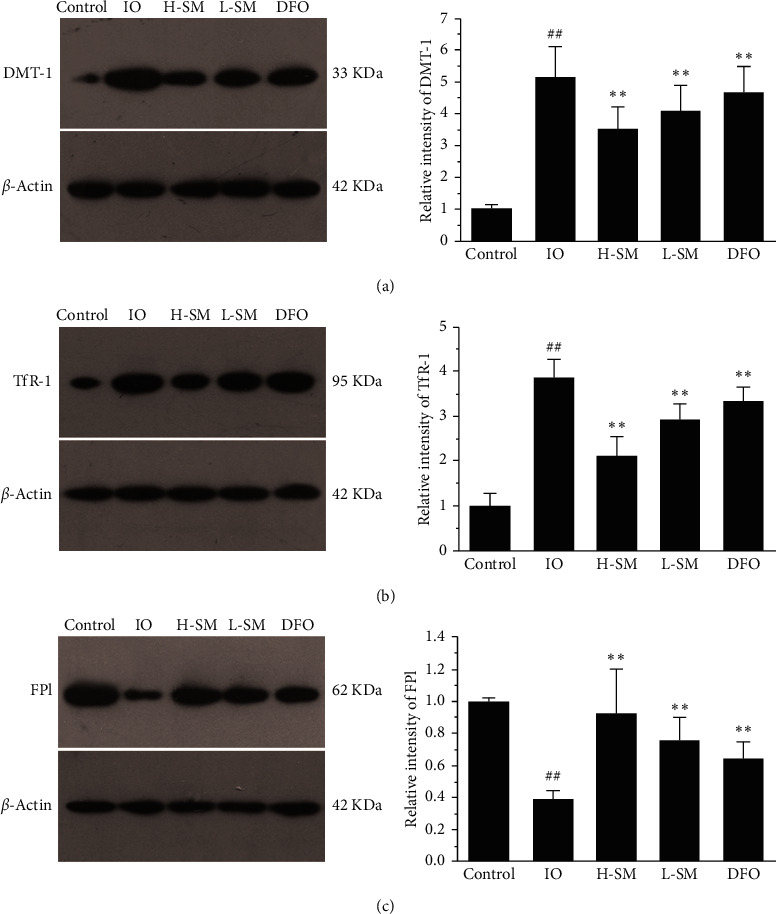
Effects of SM injection on expression levels of DMT-1, TfR-1, and FP1 in rat hearts assessed using Western blot analysis. Relative intensities were calculated by normalization to control in each group. Values are mean ± SEM. ^##^*P* < .01 vs. control group; ^*∗∗*^*P* < .01 vs. IO group.

As shown in Figure 7, the expression levels of iron uptake-related proteins DMT-1 and TfR-1 were upregulated in IO conditions, which contributed to increasing iron uptake. Besides, the iron exporter FP1 was downregulated, which reduced the iron export. The overall result was increasing the accumulation of iron in the cardiomyocytes. However, compared with the IO group, the expression levels of DMT-1 and TfR-1 were decreased and the FP1 was increased. The results indicated that SM injection could reduce the iron uptake and increase the iron export, which resulted in decreasing cardiac iron accumulation in IO conditions.

## 4. Discussion

*Salvia miltiorrhiza* (SM), known as Danshen, is a traditional Chinese medicine used for inducing blood circulation and removing blood stasis, and it has been used for the prevention and treatment of numerous ailments [14]. In this study, we investigated the cardioprotective effects and potential mechanisms of SM injection on cardiac dysfunction induced by IO. We found that SM injection downregulated the expression of DMT-1 and TfR-1 and upregulated FP1, which may be one of the mechanisms with reducing cardiac iron deposition and improving cardiac function under the conditions of IO.

In previous studies, we found that the protective mechanisms of SM against IO were at least in part due to decreased iron deposition and inhibition of oxidative stress [8, 9]. This study also exemplified that SM injection can inhibit oxidative injury by increasing SOD activity and decreasing MDA concentration (Figure 6). Previous research has suggested that, under IO conditions, excessive labile irons have a propensity for inducing and generating reactive oxygen species (ROS), resulting in cell oxidative damage [4, 15, 16]. This indicates that oxidative stress is secondary to intracellular IO. Therefore, reducing iron deposition may be a basic way to prevent IO.

Based on previous research, transferrin/transferrin receptor (Tf/TfR) and DMT-1 play important roles in iron uptake and absorption. One of the ways that cardiomyocytes can acquire iron is through Tf/TfR. More specifically, Tf binds 2 Fe^3+^circulating in the blood in the form of Fe_2_Tf. TfR-1, a transmembrane protein, can combine with Fe_2_Tf and form the Fe_2_Tf-TfR complex. This complex then enters myocardial cells through endocytosis [17, 18]. Uncombined iron remaining in the plasma is called non-transferrin-bound iron (NTBI). In addition, DMT-1 (SLC11A2), a metal transporter that is responsible for Fe^2+^ uptake, maintains normal heart structure and function [19]. Recent research has found that, in the endosome formed by the Fe2Tf-TfR complex through endocytosis, Fe^3+^ is released from Tf-TfR and the free Fe^3+^ is reduced to Fe^2+^ by STEAP3 (six-transmembrane epithelial antigen of prostate 3) and transported to the cytoplasm by DMT-1 or ZIP8/14 (zinc transporter protein) [18]. This current study found increased expression levels of myocardial DMT-1 and TfR-1 proteins in IO rats, but the expression of these same proteins decreased after treatment with SM (Figures 7(a) and 7(b)). Accordingly, Prussian blue staining and FAAS analysis determined that iron deposition was obviously reduced in the SM treatment group compared to the IO group (Figures 3 and 4). These results indicate that reduced expression of DMT-1 and TfR-1 and reduced iron uptake and absorption may be one of the mechanisms by which SM ameliorates cardiac iron overloaded-injury.

In addition to reducing iron uptake and absorption, increasing cellular iron export may be another effective way to decrease cardiac iron deposition. Ferroportin (FP) is a cellular iron-efflux channel protein expressed in enterocytes, macrophages, hepatocytes, and cardiomyocytes, and it is responsible for iron homeostasis [20–22]. Knocking out the FP gene of cardiomyocytes increases iron accumulation and results in dilated cardiomyopathy and early death of mice [22]. Our results in Figure 7(c) showed that expression levels of cardiac FP1 were upregulated in the SM group compared to the IO group. This means SM can induce FP1 expression to some extent, especially during IO conditions. These results suggest that upregulating FP1 expression and increasing iron export may also be a potential mechanism by which SM ameliorates cardiac IO injury.

In conclusion, this study indicated that, besides being an antioxidant, SM injection can inhibit cardiac iron deposition by inhibiting the expression of the iron uptake-related proteins DMT-1 and TfR-1 and promoting the expression of the iron excretion protein FP1. These three iron transporter-related proteins may serve as promising potential targets for new anti-IO drugs.

## Figures and Tables

**Table 1:** Effects of SM treatment on the hemodynamic parameters of iron overload rats.

Group	HR (beat/min)	LVSP (mmHg)	LVEDP (mmHg)	+dp/dt_max_ (mmHg/s)	−dp/dt_max_ (mmHg/s)
Control	357 ± 10	145 ± 5	3.89 ± 0.29	8874 ± 258	8468 ± 280
IO	361 ± 15	108 ± 5^*∗*^	6.84 ± 0.54^*∗*^	6578 ± 248^*∗*^	6802 ± 269^*∗*^
L-SM	347 ± 15	138 ± 5^*#*^	4.99 ± 0.47^*#*^	7869 ± 347^*#*^	8056 ± 269^*#*^
H-SM	343 ± 13	140 ± 4^*#*^	4.56 ± 0.32^*#*^	7914 ± 274^*#*^	8127 ± 213^*#*^
DFO	365 ± 14	134 ± 3^*#*^	5.12 ± 0.345^*#*^	7632 ± 163^*#*^	7943 ± 147^*#*^

HR: heart rate; LVSP: left ventricular systolic pressure; LVEDP: left ventricular end-diastolic pressure; ±dp/dt_max_: maximum ascending and descending rates of left ventricular pressure. Results are expressed as the mean ± SEM. ^*∗*^*P* < .05 vs. control group; ^#^*P* < .05 vs. iron overload (IO) group.

## Data Availability

The tiff data used to support the findings of this study are included within the article.
